# Inoculation is more effective for main crops than for preceding cover crops and does not affect the arbuscular mycorrhizal community in no-till vegetable systems

**DOI:** 10.1007/s00572-026-01255-6

**Published:** 2026-04-10

**Authors:** Clarissa Castoldi Facco, Emanuela Pille da Silva, Juliana Aparecida dos Santos, Vitória dos Santos Alves, Leonardo Khaoê Giovanetti, Emanueli Marchioro, Anabel González Hernández, Cláudio Roberto Fonsêca Sousa Soares, Jucinei José Comin, Paulo Emílio Lovato

**Affiliations:** 1 Departamento de Engenharia Rural, Universidade Federal de Santa Catarina (UFSC), Florianópolis, Santa Catarina Brazil; 2 Centro de Pesquisa Pluridisciplinar em Química, Biologia e Agricultura - CPQBA, Universidade de Campinas – UNICAMP, Campinas, São Paulo, Brasil; 3 Departamento de Microbiologia Parasitologia e Imunologia, Universidade Federal de Santa Catarina (UFSC), Florianópolis, Santa Catarina Brazil

**Keywords:** Arbuscular mycorrhizal fungi, *Abelmoschus esculents*, *Avena strigosa*, *Phaseolus vulgaris*, *Zea mays*, Soil conservation management system

## Abstract

Arbuscular mycorrhizal fungi (AMF) establish symbioses with most terrestrial plants, enhancing nutrient uptake, stress tolerance, and soil quality. We evaluated the use of *Rhizophagus irregularis*-based inoculants in cover crops and subsequent commercial crops in a no-till system and assessed their effects on soil biological attributes and crop yield. Three on-farm field experiments in different locations were conducted with common bean, okra, and corn cultivated after the winter cover crops. The assays were arranged in a randomized complete block design with a split-plot scheme and four replicates, with cover crop inoculation (black oats) as the main factor and crop inoculation in the subplot. The treatments included (i) inoculation in the cover crop and no inoculation in the main crop; (ii) no inoculation in the cover crop and inoculation in the main crop; (iii) inoculation in both crops; and (iv) no inoculation in either crop. Soil acid phosphatase activity, fluorescein diacetate hydrolysis, AMF spore density, and crop yield were measured at the end of each cycle, and the AMF community composition was characterized. The AMF inoculant increased the soil quality indicators (acid phosphatase activity and fluorescein diacetate hydrolysis). Inoculation of the cover crops did not replace the beneficial effects of direct inoculation on the yields of beans, okra, or corn. The results of the experiments in locations with different soils revealed that, overall, direct crop inoculation increased the yields of bean, okra, and corn by 30%, 33%, and 19%, respectively, and intensified soil enzyme activity, with no detectable effects on soil AMF community diversity or structure.

**Supplementary Information:**

The online version contains supplementary material available at 10.1007/s00572-026-01255-6.

## Introduction

Agricultural production must increase primarily through yield improvements in cultivated areas, offering economic gains while preserving environmental resources and meeting global demands for sustainable systems. Biologically based inputs, such as inoculants, increase plant growth via microorganisms (Brasil 2011), providing an environmentally safe and economically viable strategy. In terms of arbuscular mycorrhizal fungal (AMF) symbioses, mutualistic associations occur in ~ 74% of terrestrial plants, including major crops (van der Heijden et al. 2015). Plants supply carbon to fungi, which in turn enhances nutrient uptake, stress tolerance, and soil quality (Smith and Read 2008). AMF improve nutrient acquisition in synergy with other beneficial microbes, with effects on carbon cycling (Larimer et al. 2014; Kobae 2019; Mei et al. 2022; Shukla et al. 2025).

Despite their known benefits, large-scale AMF inoculants are constrained by fungal biotrophy, which limits supply and increases costs. Commercial applications of AMF inoculants in Brazil began in 2018, primarily in maize and soybean (Stoffel et al. 2020a, b), and the application of AMF inoculants is still concentrated predominantly in grain crops. While numerous studies have demonstrated the benefits of inoculant application in agricultural systems, the effects of inoculants on native soil microbial communities remain uncertain because of the complex interactions between introduced microorganisms and the resident microbiota adapted to local conditions (Wang et al. 2021; Hart et al. 2018). Although inoculation may cause transient shifts in fungal community composition, microbial functional redundancy and synergistic interactions among microorganisms may mitigate potential negative impacts, maintaining or even enhancing overall system functionality (Trabelsi and Mhamdi 2013; Basiru and Hijri 2022; Zhang et al. 2022).

In intensively managed agricultural systems, introduced AMF species tend to establish more readily and achieve higher success rates in soils where native AMF diversity is low; conversely, in conservation-oriented systems, such as no-till farming, higher native microbial richness may reduce inoculation effects (Basiru and Hijri 2022). There is a need to assess the medium- and long-term effects of AMF inoculation in specific settings, such as no-till vegetable systems, which combine permanent soil cover, crop rotation, and minimal soil disturbance to improve soil health and system resilience (Mafra et al. 2019). Cover crops may act as hosts and multipliers of introduced AMF, enhancing subsequent crop performance while minimizing direct inoculation needs (Buysens et al. 2016; Rivera et al. 2020). This study evaluates how an AMF inoculant containing *R. irregularis*, applied to cover crops and economic crops, affects plant yield and soil biological attributes.

## Materials and methods

Three on-farm field experiments were performed in southern Brazil (Köppen’s Cfa humid subtropical climate) to assess the effects of *Rhizophagus irregularis* inoculation on cover crops and/or succeeding vegetable crops and its implications for crop performance and soil biological attributes. All sites were managed under no-tillage, and the details of each experimental area are provided in Table S1, Fig. S1, and Fig. S2. Prior to the experiments, composite soil samples were collected for chemical characterization (Table S2). Liming and fertilization were performed according to regional recommendations (CQFS-RS/SC 2016).

Field experiments were arranged in a randomized complete block design with a split-plot scheme and four replicates. Black oats (*Avena strigosa* Schreb. ), which were sown as a cover crop, received or were not inoculated in the main plots by broadcasting 160, 150, and 90 kg ha⁻¹ of oat seeds, followed by common beans, okra, or corn, which also received or did not receive AMF inoculation. In the corn production assay, black oats were intercropped with vetch (*Vicia sativa* L., 70 kg seeds ha⁻¹). The AMF fungus *R. irregularis* inoculant, containing 2.5 10^− 3^ spores g⁻¹, was applied at 50 g ha⁻¹ as recommended by the manufacturer, first on the seeds of black oats, and the following treatments were carried out: inoculated (Oat+) or noninoculated (Oat-). At flowering, the black oats were rolled down, and the main plots were divided into subplots at the establishment of the main crops (cash crops), which had the inoculant applied on their seeds, in the same way as the previous inoculation of the cover crop (Fig. S2). This resulted in four treatments: Oat+/noninoculated main crop (Bean-; Okra-; or Corn-), Oat+/inoculated main crop (Bean+; Okra+; or Corn+), Oat-/inoculated main crop (Bean+; Okra+; or Corn+), and Oat-/noninoculated main crop (Bean-; Okra-; or Corn-).

Sowing was carried out in six (common beans) or four (okra and corn) rows, with lengths of 5, 15, and 10 m. The spacing between rows was 0.30, 1.0 and 0.90 m, and that between plants was 0.30, 0.40 and 0.30 m for common beans, okra, and corn, respectively. During the flowering of the main crop, composite samples (10 subsamples) were collected from the 0–10 cm soil layer. Ten plants per plot were sampled to evaluate two yield components in common beans: grain weight and the number of pods per plant. In okra, representative plants from the two central rows of each plot were sampled weekly over four consecutive weeks. Fruit weight and length were recorded as yield components. For maize, 20 plants per plot were sampled, and the yield was estimated from the dry mass of the ear.

Samples for biological analyses were stored at 4 °C until processing and at -80 °C for the molecular analyses of AMF communities. AMF spores were extracted by wet sieving (Gerdemann and Nicolson 1963) from 50-cm³ samples and counted. Acid phosphatase activity (AP) was determined by quantifying *p*-nitrophenol released after the hydrolysis of *p*-nitrophenyl phosphate (Tabatabai and Bremner 1969; Eivazi and Tabatabai 1977). Fluorescein diacetate (FDA) hydrolysis was measured following the methods of Schnurer and Rosswall (1982). Soil DNA was extracted with a DNeasy PowerSoil Kit (QIAGEN) and quantified with a NanoDrop Lite spectrophotometer (Thermo Fisher Scientific, USA), and the 18 S rRNA gene region was amplified with nested PCR targeting AMF. The primers and amplification conditions are specified in Table S3. Indexed libraries were prepared with Nextera XT Index Primers (Illumina, USA), purified, quantified, and pooled equimolarly. Sequencing was performed on an Illumina NextSeq platform (2 × 300 bp). The raw reads were quality checked (FastQC v0.12), trimmed (Cutadapt v5. ), and processed using DADA2 v1.22.0 in R for filtering, dereplication, error correction, and chimera removal. Taxonomy was assigned with assignTaxonomy against the 18 S rRNA reference and MaarjAM databases (Öpik et al. 2010). Nontarget sequences were excluded.

Data on acid phosphatase activity, FDA hydrolysis, spore density, and crop yield were tested for variance homogeneity (Bartlett test) and analyzed by ANOVA at the 5% probability level; significant effects were compared using Tukey’s test. Amplicon sequence variant (ASV) counts were normalized using Hellinger transformation (vegan v2.6-2; Oksanen et al. 2022) to account for sequencing depth without rarefaction (Legendre et al. 2001; McMurdie and Holmes 2014). ASV counts were aggregated at the family and genus levels using phyloseq. Counts were converted to relative abundance (%) per sample and averaged by crop and treatment. The results were visualized as stacked bar plots in ggplot2. Alpha diversity indices (Shannon and Chao1) were tested by paired Wilcoxon tests, with p values adjusted using the Benjamini–Hochberg method (FDR). Beta diversity was assessed using Bray–Curtis dissimilarity, visualized by PCoA, and tested by PERMANOVA (adonis2, 999 permutations). Pairwise PERMANOVA comparisons between treatments were performed with pairwiseAdonis (Martinez Arbizu 2020), with both false discovery rate (FDR) and Bonferroni correction applied to p values. The homogeneity of multivariate dispersions was verified with betadisper and permutest. All statistical analyses were performed in R (R Core Team 2011).

## Results

**Fig. 1:**
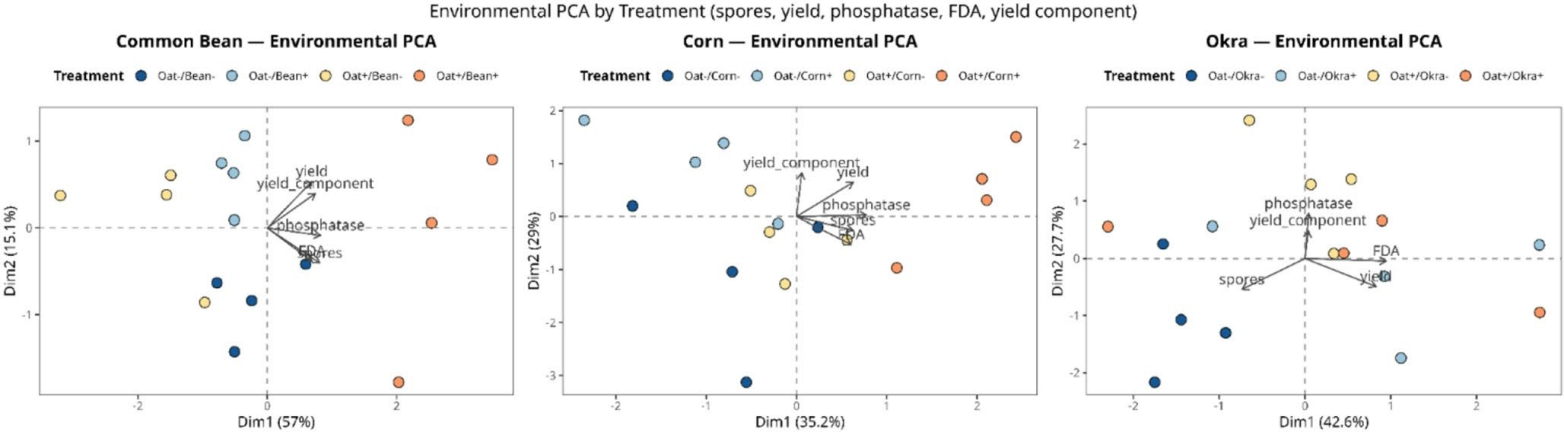
PCA of yield, AMF spore density, acid phosphatase activity, and FDA hydrolysis across the main crop and cover crop treatments. The treatments included the inoculation of black oats (Oat+) and/or the main crops (Bean+, Corn+, and Okra+). Vectors represent variable loadings; points correspond to replicate plots. Dim1 and Dim2 percentages indicate the proportion of explained variance in each dimension

Principal component analyses (PCA) provided an integrated overview of the variables affected by inoculation across crops (Fig. 1). The first two principal components explained 64–69% of the total variance, capturing the main gradients of biological and agronomic responses. Treatments with dual inoculation (Oat+/Crop+) were mostly positioned along the positive side of the first axis and were strongly associated with increased soil biological activity and increased yield. In contrast, the noninoculated treatments clustered on the negative side, reflecting lower microbial activity and reduced yield.

The multivariate patterns converged with the univariate ANOVA results, confirming that AMF inoculation enhanced soil biological attributes (Fig. S3) and crop yield (Fig. S4). ANOVA confirmed the significant effects of AMF inoculation on all crops. In common beans, compared with Oat+/Bean- treatment, double inoculation increased acid phosphatase activity and spore density by 49% and 128%, respectively, and increased the pod number by 29%. The highest yield was observed when the main crop was inoculated, regardless of prior cover crop inoculation, resulting in a 30% increase compared with that of Bean-. In okra, inoculation increased AP and FDA activities, with black oat inoculation increasing AP activity by 19% relative to that in oat– and okra inoculation increasing FDA activity by 27% compared to that in okra–. Spore density was 17% greater in noninoculated black oats, and pod length slightly decreased (6%) with dual inoculation. The highest yield was observed when the main crop was inoculated, regardless of the previous crop inoculation, with a 33% increase in relation to that of okra-. Compared with Oat+/Corn–, cover crop inoculation increased AP activity and FDA activity by 24% and 43%, respectively, while compared with Oat+/Corn– inoculation, dual inoculation increased spore density by 34%. Ear dry mass was greater when the main crop was inoculated, resulting in a 19% increase relative to that of corn–.

**Fig. 2:**
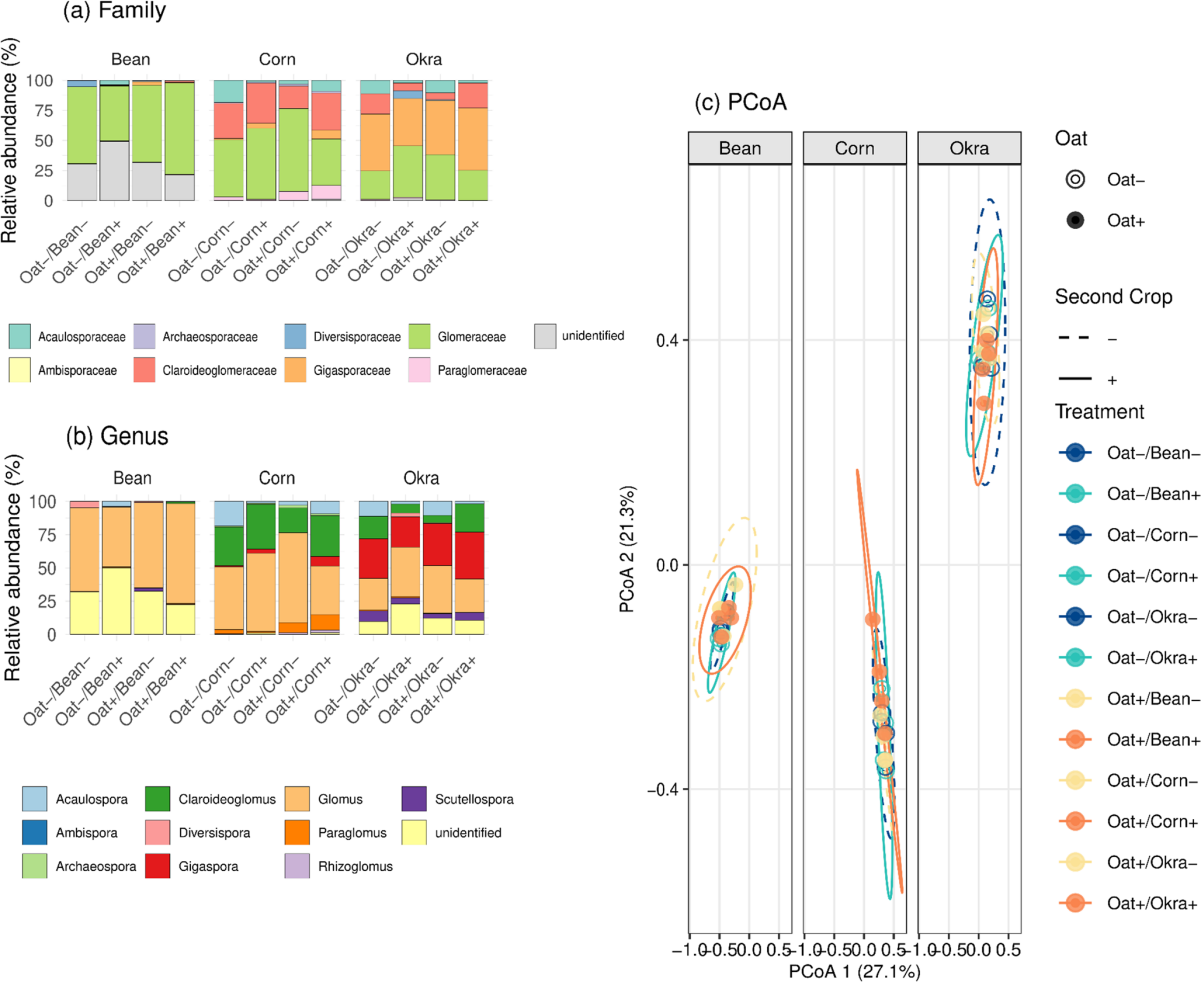
AMF community composition and diversity in response to oat cover crop management. **a**–**b** Relative abundance of dominant arbuscular mycorrhizal fungal families and genera across treatments. **c** PCoA ordination showing the distribution of samples in ordination space based on the Bray–Curtis dissimilarity for each main crop. Ellipses represent 95% confidence intervals. Treatment labels: Oat+/- (cover crop presence/absence), Bean/Corn/Okra (main crop type), +/- (inoculation status)

The AMF community was largely dominated, at the family level, by Glomeraceae, comprising more than 60% of the sequences in the common bean trial and more than 50% in the corn assay (Fig. 2). In contrast, the okra trial showed a more balanced composition, with Glomeraceae and Gigasporaceae comprising approximately 30% and 40% of the community, respectively. Approximately 30% of the sequences in the common bean trial remained unclassified at the family level. At the genus level, *Glomus* prevailed in common bean and corn systems, accounting for more than half of the community (Fig. 2). In okra, *Gigaspora* and *Glomus* occurred in similar proportions, each contributing approximately 30% of the sequences.

Community richness varied across the cropping systems, with mean ASV counts of 212, 250, and 256 for common bean, okra, and corn, respectively (Table S4). A Venn diagram (Fig. S5) shows differences in the AMF community assemblages among the treatments evaluated within each site, with only 27–38% of the ASVs shared and the majority being treatment specific. Pairwise comparisons of alpha diversity (Shannon and Chao1 indices) revealed no significant differences in the AMF community structure among the inoculation treatments within each crop (Table S5). Across all the systems (Oat + Bean, Oat + Okra, and Oat + Corn), *R. irregularis* inoculation did not significantly alter the AMF community composition at the ASV level (PERMANOVA, *p* > 0.082). Inoculation effects on either the cover or main crops were likewise nonsignificant. Dispersion homogeneity was confirmed in all cases (betadisper, *p* > 0.19), indicating that the lack of effects was not confounded by differences in variance. Principal coordinate analysis (PCoA) based on Bray–Curtis dissimilarities revealed no clear separation among the inoculation treatments within each crop (Fig. 2). Similarly, pairwise PERMANOVA comparisons revealed no significant differences in the AMF community structure between treatment pairs (Table S6).

## Discussion

Our results demonstrate that yield gains were greater when AMF were applied directly to the main crop than to the cover crop. These findings contrast with reports suggesting that cover crop inoculation increases AMF propagule abundance and subsequently benefits the following crop (Buysens et al. 2016; Rivera et al. 2020; Espinosa et al. 2023). In our experiments, direct inoculation resulted in substantial yield increases—30% in common beans, 33% in okra, and 19% in maize— indicating a more pronounced and immediate effect on crop performance. *R. irregularis*, a generalist species used in most commercial inoculants (Thomsen and Hart 2018), performed well across a broad range of pH values (4.4–7.1) in our assays, indicating ecological plasticity. However, responses to AMF application are context dependent, as demonstrated by Lahijanian et al. (2025), with maize inoculated with AMF and bacteria in maize fields that had or had not received cover crops. The magnitude of the response depends on host species, nutrient availability, and the resident AMF community, highlighting that propagule availability and edaphic compatibility are key factors for successful inoculation in no-till vegetable systems. Multivariate analyses indicated that in three different soils with different plant species, the inoculant increased yield and affected soil attributes as well.

AMF inoculation affects soil microbiological attributes, particularly enzyme activity. Acid phosphatase activity increased, suggesting a potential increase in phosphorus mobilization and soil functional quality. The formation of the mycorrhizosphere—an interface surrounding external fungal hyphae where carbon is exuded—may contribute to this response by stimulating microbial communities involved in nutrient cycling (Hartmann et al. 2009). Previous studies have indicated that the combined use of cover crops and AMF may stimulate phosphatase activity and promote organic phosphorus (Po) hydrolysis, partly through shifting bacterial communities, including those harboring alkaline phosphatase (alp) genes, which have been reported to increase organic P mineralization and potentially increase P availability (Cui et al. 2015; Zhang et al. 2018; Mitra et al. 2023; Garcia et al. 2025; Pu et al. 2025). In agreement with these findings, increased phosphatase activity was observed under cover-crop-based management when AMF were applied (Ferreira et al. 2024), supporting the possible role of inoculation in enhancing P mobilization pathways. In long-term no-tillage systems, the accumulation of soil organic matter from continuous residue inputs can increase biological activity, soil aggregation, and water retention (Comin et al. 2024). Such conditions can create a favorable environment for AMF-associated processes, helping to support nutrient cycling and plant performance. Overall, soil enzymatic activity, estimated by FDA hydrolysis, also increased with inoculation in most of our experiments, suggesting that AMF stimulates soil biological function.

Although AMF inoculation stimulated soil enzymatic activity, it did not affect native AMF richness or community composition, indicating functional shifts without detectable structural changes in the community. This decoupling between community composition and function suggests that inoculation effects were likely transient but sufficient to influence nutrient cycling and plant performance. Importantly, these responses occurred under no-till vegetable production, a system typically characterized by relatively stable and diverse microbial communities. While AMF inoculation is often considered more effective in soils with reduced native diversity (Basiru and Hijri 2022; Kinnunen et al. 2016), our findings indicate that targeted inoculation can provide agronomic benefits even in conservation systems, likely by enhancing early root–fungus interactions rather than by reshaping the resident AMF community.

Fungal communities, closely associated with plants, are more resilient to disturbances than bacteria are, which respond more readily to inoculation (Cornell et al. 2021). Intensive agricultural practices, including high chemical inputs and mechanical disturbances, can reduce the abundance of native species, making those areas more prone to respond to AMF inoculation (Brígido et al. 2017). In our study, the AMF communities were dominated by *Glomus*, *Claroideoglomus*, *Gigaspora*, and *Acaulospora*. The introduction of *R. irregularis* did not affect native community richness or composition, indicating that, in general, microbial inoculation effects are generally transient, as diverse native communities are resilient and restrict the persistence of introduced species (Wang et al. 2021).

Different functional strategies among AMF families affect nutrient acquisition, soil structure, and microbial dynamics. Glomeraceae species are often characterized by rapid growth, high sporulation rates, and greater tolerance to disturbance, which may contribute to their frequent dominance in agricultural systems (Van der Heyde et al. 2017). In contrast, members of the Gigasporaceae typically allocate more resources to extensive extraradical mycelia, resulting in greater tolerance to chemical disturbances but lower resistance to mechanical and aerial stress. Acaulosporaceae species are generally associated with slower growth, lower root colonization rates, and dormant spores, with greater tolerance to mechanical disturbances. Even when introduced microorganisms do not establish long-term dominance within native communities, they may stimulate early root development and modulate plant metabolism, producing functionally meaningful effects on root architecture and associated microbial interactions (Bashan 1999).

In diverse systems such as the NTVS, the impact of inoculation tends to have a limited effect on indigenous communities, as observed in our trials, but can complement the functions of native AMF, increasing yield. Commercial AMF inoculants are generally developed to complement or enhance functions that native communities may not fully provide to crops. For effectiveness, however, introduced strains must successfully establish and persist in roots and soil, at least during the intended period, while interacting with indigenous fungal species. Effective inoculants are selected for rapid proliferation, resource efficiency, and competitive ability, as seen with *R. irregularis* (Thomsen and Hart 2018), which likely contributed to the observed yield gains in this work. These aspects help explain why the outcomes of inoculation often vary depending on the composition and resilience of the indigenous community. In addition, *R. irregularis* is recognized for its high root colonization rate (Werner and Kiers 2015). Future studies using root-focused amplicon analyses might clarify inoculant colonization dynamics relative to indigenous AMF.

## Conclusions

The yields of common beans, okra, and corn increased with AMF inoculation, but inoculation with the preceding cover crop did not affect the yield of the subsequent main crops.

The *R. irregularis*-based inoculant enhanced soil biological activity, as evidenced by increased acid phosphatase activity and fluorescein diacetate (FDA) hydrolysis in no-tillage vegetable production systems.

AMF inoculation did not alter the soil AMF community richness or structure.

## Supplementary Information

Below is the link to the electronic supplementary material.

Supplementary Material 1

## Data Availability

No datasets were generated or analysed during the current study.
