# Supplementary material for: Inoculation is more effective for main crops than for preceding cover crops and does not affect the arbuscular mycorrhizal community in no-till vegetable systems
Source: Mycorrhiza. 2026 Apr 10;36(2):13. doi: 10.1007/s00572-026-01255-6 (PMC13068752; doi:10.1007/s00572-026-01255-6)
Supplement: Supplementary file 1 — Supplementary Material 1 [file 572_2026_1255_MOESM1_ESM.docx]

**Supplementary material**

Clarissa Castoldi Facco^1^, Emanuela Pille da Silva¹, Juliana Aparecida dos Santos^2^, Vitória dos Santos Alves¹, Leonardo Khaoê Giovanetti^1^, Emanueli Marchioro^3^, Anabel González Hernández^3^, Cláudio Roberto Fonsêca Sousa Soares^3^, Jucinei José Comin^1^, Paulo Emílio Lovato^1*^

^1^ Universidade Federal de Santa Catarina (UFSC), Departamento de Engenharia Rural, Florianópolis, Santa Catarina, Brazil

^2^Universidade de Campinas – UNICAMP, Centro de Pesquisa Pluridisciplinar em Química, Biologia e Agricultura-CPQBA, Campinas, São Paulo, Brasil.

^3^Universidade Federal de Santa Catarina (UFSC), Departamento de Microbiologia Parasitologia e Imunologia, Florianópolis, Santa Catarina, Brazil

*[paulo.lovato@ufsc.br](mailto:paulo.lovato@ufsc.br); manupille@gmail.com

**Table S1** Location and general characteristics of the assays assessing the effect of *R. irregularis* in no-till vegetable systems (NTVS) in Southern Brazil.

| **Municipality** | **Coordinates/**  **Elevation (m)** | **Temperature (°C)¹** | **Precipitation (mm)¹** | **Seeding Rates of Cover Crops** | **Plant species** | **Period of cultivation** |
| --- | --- | --- | --- | --- | --- | --- |
| Águas Mornas | 27°43'54"S  8°57'00"W  287 | 20.8² | 1788 | 160 kg ha⁻¹ | Black oats and common bean (*Phaseolus vulgaris)* | 04/2023 - 02/2024 |
| Antônio Carlos | 27°31'24"S 48°48'12"W  26 | 21.0³ | 1861 | 150 kg ha⁻¹ | Black oats and okra  (*Abelmoschus esculents*) | 05/2023 - 02/2024 |
| Santa Rosa de Lima | 28°02'59"S 49°09'46"W  371 | 19.3^4^ | 1823 | 90 kg ha⁻¹+70 kg ha⁻¹ | Black oat + vetch (*Vicia sativa)* and corn (*Zea mays*) | 05/2023 - 02/2024 |

¹ Values recorded during the test period. ² Data obtained at the meteorological station 2242-Águas Mornas - Beira Rio- EPAGRI. ³ Data obtained at the meteorological station 2383-Antonio Carlos - Bairro Usina - EPAGRI; ^4^ Data obtained at the meteorological station 2255-Rio Fortuna - Rio Café - - EPAGRI.

**Table S2 S**oil chemical analysis from no-till vegetable system (NTVS) assays with black oat and bean, okra, or corn inoculated with *R. irregularis*, Santa Catarina State, Brazil.

| **Municipality/Crop** | **Depth**  **(cm)** | **P³** | **K³** | **Al²** | **Ca²** | **Mg²** | **CEC pH7.0** | **BS¹** | **Clay^5^** | **pH** | **MO^4^** |
| --- | --- | --- | --- | --- | --- | --- | --- | --- | --- | --- | --- |
|  |  | mg dm³ | | cmol_c_ dm³ | | | | % | **%** | H_2_O | g kg^-1^ |
| Águas Mornas/Beans | 0-10 | 302.4 | 289 | 0 | 7.03 | 2.26 | 11.76 | 85.4 | 34 | 7.13 | 37 |
|  | 10-20 | 40 | 344.1 | 0 | 4.42 | 1.32 | 10.46 | 64.44 | 25 | 6.42 | 19.7 |
| Antônio Carlos/Okra | 0-10 | 111.25 | 109.35 | 0.2 | 3.95 | 0.72 | 9.78 | 52.31 | 22 | 5.42 | 13.2 |
|  | 10-20 | 87.62 | 90.6 | 0.05 | 3.65 | 0.62 | 8.28 | 55.12 | 23.25 | 5.47 | 9.7 |
| Santa Rosa de Lima/ Corn | 0-10 | 9.06 | 75.8 | 1.1 | 1.8 | 0.44 | 9.86 | 25.97 | 25 | 4.4 | 28.4 |
|  | 10-20 | 3 | 54.44 | 1.76 | 1.2 | 0.38 | 10.69 | 16.88 | 23.4 | 4.36 | 18.6 |

¹BS %=Base saturation; ^2^ Extracted with 1 mol L^-1^ KCl; ^3^ Extracted with Mehlich^-1^; ^4^ Walkley-Black Method; ^5^ Pipette Method.

**Table S3** PCR Primer Pairs and Conditions for Amplification of Arbuscular Mycorrhizal Fungal DNA.

| **Step** | **Primers (5’–3’)** | **Reference** | **Reaction Components**  **(25 µL Total Volume)** | **Cycling Conditions** |
| --- | --- | --- | --- | --- |
| First PCR | NS31: GAA CCC AAA CAC TTT GGT TTC C AML2: GAA CCC AAA CAC TTT GGT TTC C | Simon et al. 1992);  Lee et al. (2008) | 1 µL DNA 0.2 µM each primer 10 µL 2× PCRBio Ultra Mix (PCRBiosystems, UK) Nuclease-free water to final volume | 94 °C for 2 min;  33 cycles of: • 94 °C for 30 s • 65 °C for 30 s • 72 °C for 40 s Final extension:  • 72 °C for 10 min |
| Second PCR | AMV4.5NF: AAG CTC GTA GTT GAA TTT CG AMDGR: CCC AAC TAT CCC TAT TAA TCA T | Goll et al. (2014) | 1 µL of purified product as template Other components as in first PCR | Similar conditions to Frist PCR, except for annealing at 58 °C for 30 s over 28 cycles |

**Table S4** Amplicon sequence variant (ASV) richness across NTVS trials and inoculation treatments. Total ASVs represents the cumulative number of unique ASVs detected across all replicates per treatment (n = 4).

| **Crop** | **Treatment** | **Total Reads** | **Total ASVs** |
| --- | --- | --- | --- |
| Bean | oat +/ bean + | 484515 | 194 |
|  | oat +/ bean - | 422883 | 201 |
|  | oat -/ bean + | 475844 | 231 |
|  | oat -/ bean - | 464944 | 224 |
| Okra | oat +/ okra + | 352436 | 218 |
|  | oat +/ okra - | 318323 | 266 |
|  | oat -/ okra + | 327770 | 273 |
|  | oat -/ okra - | 330129 | 243 |
| Corn | oat +/ corn + | 445689 | 303 |
|  | oat +/ corn - | 464398 | 211 |
|  | oat -/ corn + | 442384 | 274 |
|  | oat -/ corn - | 441229 | 238 |

**Table S5** Paired comparisons of Shannon and Chao diversity indices among treatments, assessed using the paired Wilcoxon test with *p*-value adjustment by the Benjamini–Hochberg (FDR) method.

|  |  | **Shannon index** | | **Chao 1 index** | |
| --- | --- | --- | --- | --- | --- |
| **Treatment 1** | **Treatment 2** | **P-value adjusted** | **significance** | **P-value adjusted** | **significance** |
| **Oat+Bean** | | | | | |
| Oat-/Bean- | Oat-/Bean+ | 1 | ns | 0,842 | ns |
| Oat-/Bean- | Oat+/ Bean- | 1 | ns | 0,882 | ns |
| Oat-/Bean- | Oat+/Bean+ | 0,972 | ns | 0,842 | ns |
| Oat-/Bean+ | Oat+ Bean- | 1 | ns | 0,842 | ns |
| Oat-/Bean+ | Oat+/Bean+ | 0,972 | ns | 0,842 | ns |
| Oat+/Bean- | Oat+/Bean+ | 0,972 | ns | 0,882 | ns |
| **Oat+Corn** | | | | | |
| Oat-/Corn- | Oat-/Corn+ | 1 | ns | 0,885 | ns |
| Oat-/Corn- | Oat+/Corn- | 1 | ns | 0,69 | ns |
| Oat-/Corn- | Oat+/Corn+ | 1 | ns | 0,228 | ns |
| Oat-/Corn+ | Oat+/Corn- | 1 | ns | 0,228 | ns |
| Oat-/Corn+ | Oat+/Corn+ | 1 | ns | 0,823 | ns |
| Oat+/Corn- | Oat+/Corn+ | 1 | ns | 0,228 | ns |
| **Oat+Okra** | | | | | |
| Oat-/Okra- | Oat-/Okra+ | 0,729 | ns | 0,515 | ns |
| Oat-/Okra- | Oat+/Okra- | 1 | ns | 0,515 | ns |
| Oat-/Okra- | Oat+/Okra+ | 0,729 | ns | 0,673 | ns |
| Oat-/Okra+ | Oat+/Okra- | 1 | ns | 1 | ns |
| Oat-/Okra+ | Oat+/Okra+ | 0,343 | ns | 0,244 | ns |
| Oat+/Okra- | Oat+/Okra+ | 0,6 | ns | 0,244 | ns |

**Table S6.** Pairwise comparisons between treatments using PERMANOVA (adonis2) based on Bray-Curtis distance.

| **Treatment 1** | **Treatment 2** | **R²** | **F** | **p-value** |
| --- | --- | --- | --- | --- |
| **Oat +bean** | | | | |
| Oat-/Bean- | Oat-/Bean+ | 0.1235 | 0.8456 | 0.617 |
| Oat-/Bean- | Oat+/ Bean- | 0.1254 | 0.8603 | 0.507 |
| Oat-/Bean- | Oat+/Bean+ | 0.1092 | 0.7352 | 0.821 |
| Oat-/Bean+ | Oat+ Bean- | 0.0907 | 0.5982 | 0.917 |
| Oat-/Bean+ | Oat+/Bean+ | 0.2125 | 1.6193 | 0.082 |
| Oat+/Bean- | Oat+/Bean+ | 0.1639 | 1.1759 | 0.288 |
| **Oat + Corn** | | | | |
| Oat-/Corn- | Oat-/Corn+ | 0.1205 | 0.8223 | 0.795 |
| Oat-/Corn- | Oat+/Corn- | 0.1304 | 0.8996 | 0.604 |
| Oat-/Corn- | Oat+/Corn+ | 0.1488 | 1.0490 | 0.423 |
| Oat-/Corn+ | Oat+/Corn- | 0.1638 | 1.1753 | 0.259 |
| Oat-/Corn+ | Oat+/Corn+ | 0.1239 | 0.8482 | 0.766 |
| Oat+/Corn- | Oat+/Corn+ | 0.1682 | 1.2132 | 0.275 |
| **Oat + Okra** | | | | |
| Oat-/Okra- | Oat-/Okra+ | 0.1289 | 0.8880 | 0.622 |
| Oat-/Okra- | Oat+/Okra- | 0.0819 | 0.5351 | 0.971 |
| Oat-/Okra- | Oat+/Okra+ | 0.1117 | 0.7546 | 0.927 |
| Oat-/Okra+ | Oat+/Okra- | 0.1215 | 0.8297 | 0.727 |
| Oat-/Okra+ | Oat+/Okra+ | 0.1390 | 0.9685 | 0.587 |
| Oat+/Okra- | Oat+/Okra+ | 0.0887 | 0.5841 | 0.974 |

**
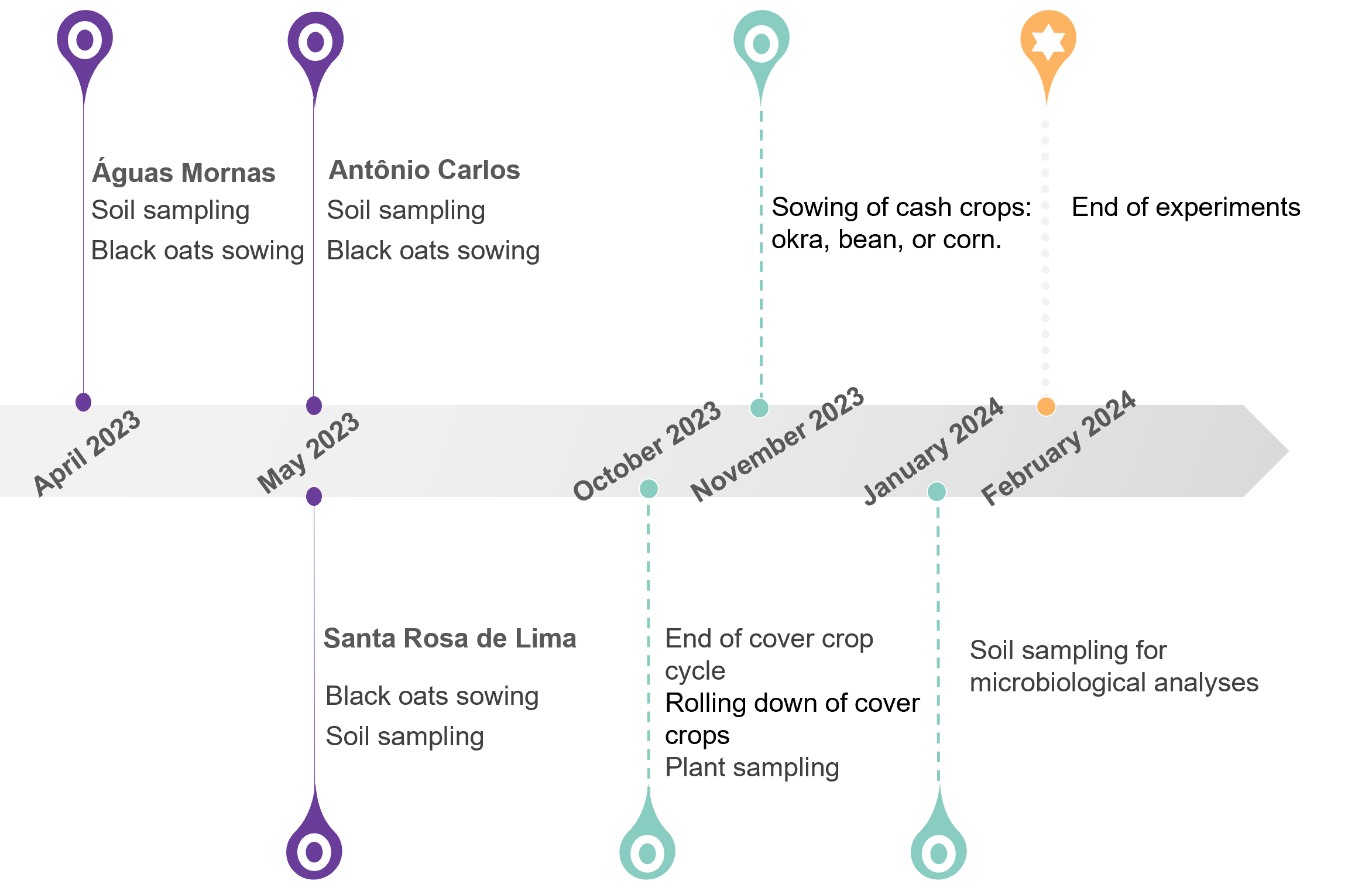
**

**Fig S1** Timeline of the field experiments showing the crop sequence from black oats (*Avena strigosa*) to common bean, corn, and okra, with AMF (*R. irregularis*) inoculation in black oat and/or the subsequent crop.


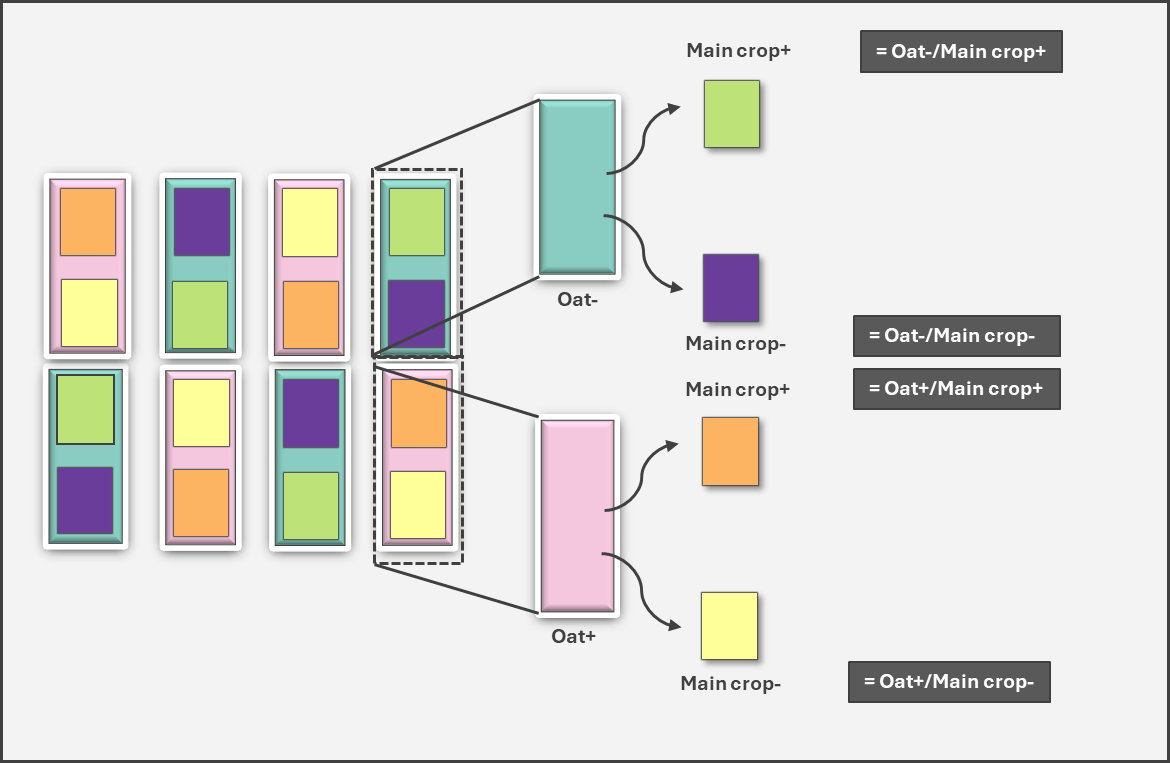


**Fig S2** Schematic overview of the field experiment showing the crop sequence with black oats (*A. strigosa*) and subsequent crops (common bean, corn, and okra). Treatments involved inoculation with or without AMF (*R. irregularis*) in the black oat and/or the following crop (Oat–/Crop–, Oat+/Crop–, Oat–/Crop+, Oat+/Crop+). The experiment followed a randomized complete block design in a split-plot scheme across different locations to assess soil biology attributes and AMF community as well as crop performance.


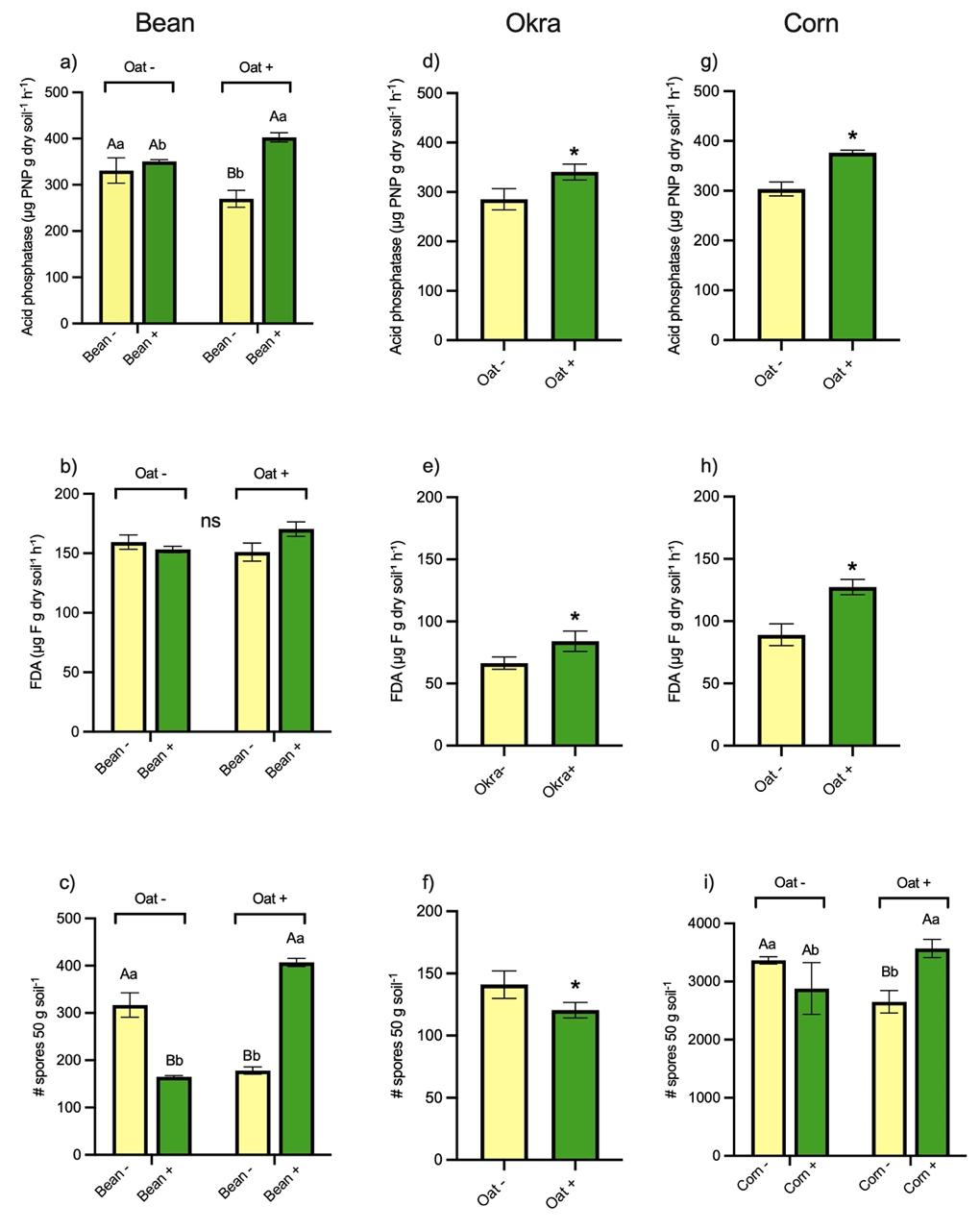


**Fig S3** Soil acid phosphatase activity (a, d, g), fluorescein diacetate hydrolysis (FDA) (b, e, h), and arbuscular mycorrhizal fungi (AMF) spore density (c, f, i) obtained from a split-plot experiment conducted with common bean, okra, and corn. Each crop was cultivated after black oat that was either inoculated (+) or not (−) with AMF. In each main plot (Oat inoculation), the subplots consisted of crops inoculated (+) or not (−) with AMF. Treatments were: Oat+/non-inoculated bean (Bean−), Oat+/inoculated bean (Bean+), Oat−/Bean−, and Oat−/Bean+; Oat+/non-inoculated okra (Okra−), Oat+/inoculated okra (Okra+), Oat−/Okra−, and Oat−/Okra+; Oat+/non-inoculated corn (Corn−), Oat+/inoculated corn (Corn+), Oat−/Corn−, and Oat−/Corn+. “ns” indicates no significant difference by the ANOVA F-test. “*” indicates significant differences for individual factors according to Tukey’s test (p ≤ 0.05). Different letters denote significant differences among combined treatments according to Tukey’s test (p ≤ 0.05). Uppercase letters compare crop inoculation treatments within the same black oat inoculation level, while lowercase letters compare them across different black oat inoculation levels.


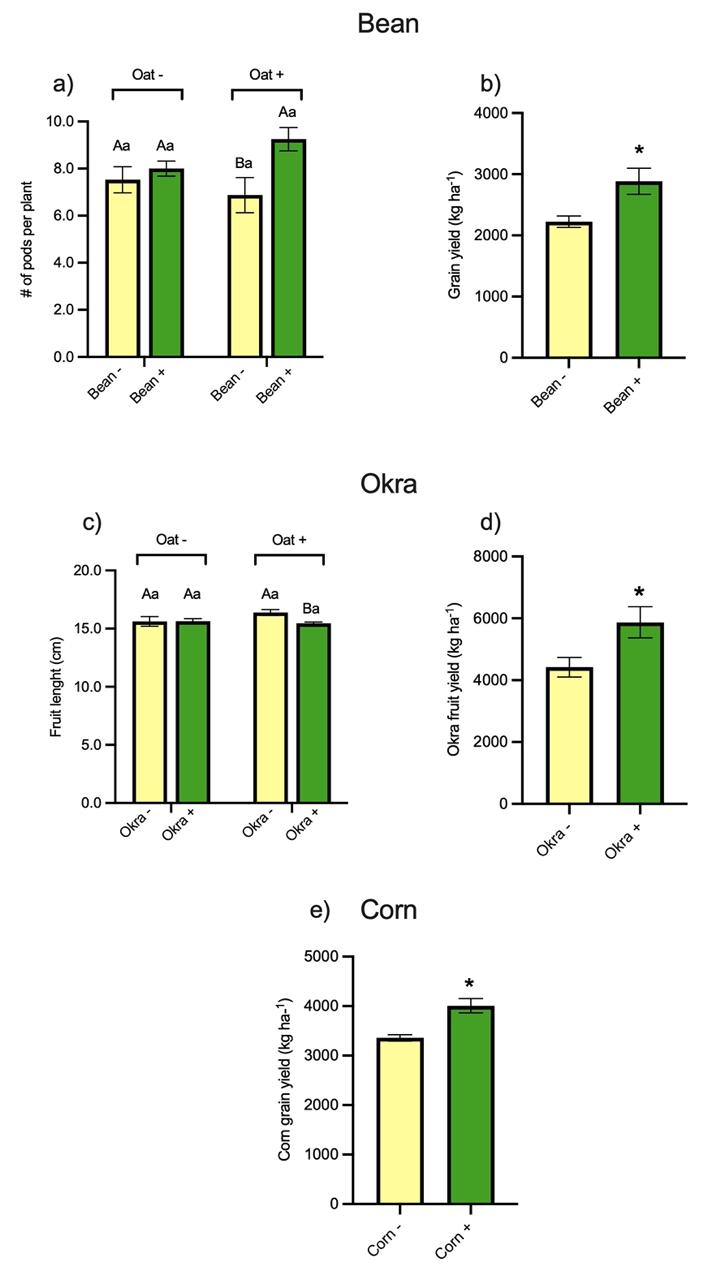


**Fig S4** Number of pods, grain yield of common bean (a e b), fruit length and fruit yield of okra (c e d), and yield of corn (e) obtained from a split-plot experiment conducted with common bean, okra, and corn. Each crop was cultivated after black oat that was either inoculated (+) or not (−) with AMF. In each main plot (black oat inoculation), the subplots consisted of crops inoculated (+) or not (−) with AMF. Treatments were: Oat+/non-inoculated bean (Bean−), Oat+/inoculated bean (Bean+), Oat−/Bean−, and Oat−/Bean+; Oat+/non-inoculated okra (Okra−), Oat+/inoculated okra (Okra+), Oat−/Okra−, and Oat−/Okra+; Oat+/non-inoculated corn (Corn−), Oat+/inoculated corn (Corn+), Oat−/Corn−, and Oat−/Corn+. “ns” indicates no significant difference by the ANOVA F-test. “*” indicates significant differences for individual factors according to Tukey’s test (p ≤ 0.05). Different letters denote significant differences among combined treatments according to Tukey’s test (p ≤ 0.05). Uppercase letters compare crop inoculation treatments within the same black oat inoculation level, while lowercase letters compare them across different black oat inoculation levels.

**
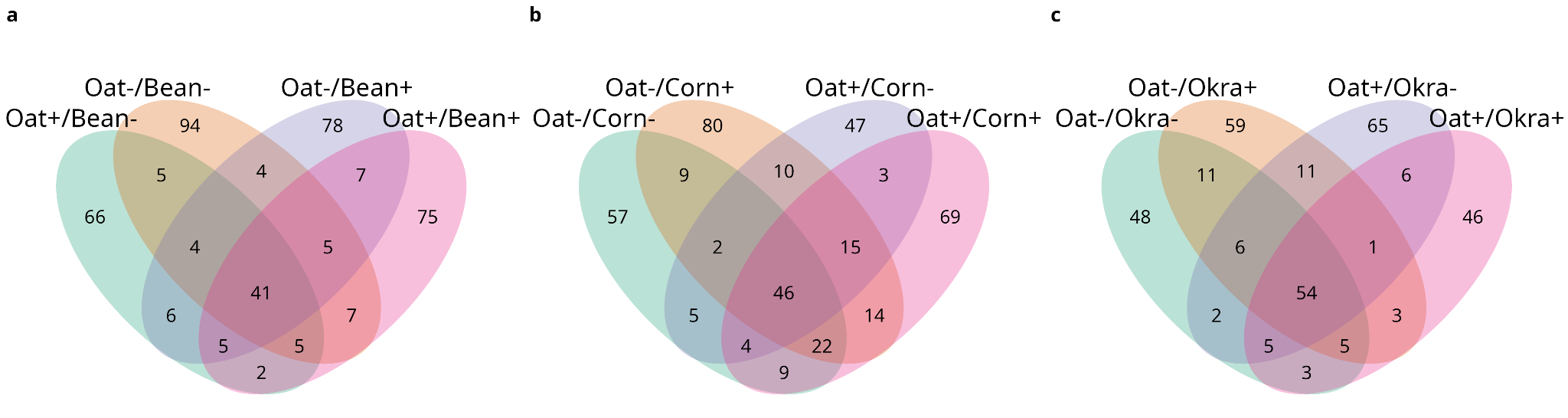
**

**Fig S5** Venn diagram illustrating the overlap of arbuscular mycorrhizal fungi (AMF) Amplicon Sequence Variants (ASVs) detected across the different treatments evaluated in each assay (a) bean, (b) corn) and (c) okra. Numbers in the intersections represent ASVs numbers shared among treatments, while values exclusive to each set indicate ASVs unique to a specific treatment. The analysis was based on the 18S rRNA region amplified and sequenced using next-generation sequencing (NGS).
